# Vaccination with *Leishmania infantum* Acidic Ribosomal P0 but Not with Nucleosomal Histones Proteins Controls *Leishmania infantum* Infection in Hamsters

**DOI:** 10.1371/journal.pntd.0003490

**Published:** 2015-02-02

**Authors:** Lais Pereira, Melissa Abbehusen, Clarissa Teixeira, Jurema Cunha, Ivan P. Nascimento, Kyioshi Fukutani, Washington dos-Santos, Aldina Barral, Camila Indiani de Oliveira, Manoel Barral-Netto, Manoel Soto, Cláudia Ida Brodskyn

**Affiliations:** 1 Centro de Pesquisa Gonçalo Moniz, FIOCRUZ-BA, Bahia, Brazil; 2 Centro de Biotecnologia, Instituto Butantan, São Paulo, Brazil; 3 Faculdade de Medicina, Universidade Federal da Bahia, Bahia, Brazil; 4 Instituto de Investigação em Imunologia, São Paulo, Brazil; 5 Centro de Biología Molecular "Severo Ochoa," Universidad Autónoma de Madrid, Madrid, Spain; 6 Instituto de Ciências da Saúde, Universidade Federal da Bahia, Bahia, Brazil; Institut Pasteur de Tunis, TUNISIA

## Abstract

**Background:**

Several intracellular *Leishmania* antigens have been identified in order to find a potential vaccine capable of conferring long lasting protection against *Leishmania* infection. Histones and Acid Ribosomal proteins are already known to induce an effective immune response and have successfully been tested in the cutaneous leishmaniasis mouse model. Here, we investigate the protective ability of *L. infantum* nucleosomal histones (HIS) and ribosomal acidic protein P0 (LiP0) against *L. infantum* infection in the hamster model of visceral leishmaniasis using two different strategies: homologous (plasmid DNA only) or heterologous immunization (plasmid DNA plus recombinant protein and adjuvant).

**Methodology/Principal Findings:**

Immunization with both antigens using the heterologous strategy presented a high antibody production level while the homologous strategy immunized group showed predominantly a cellular immune response with parasite load reduction. The pcDNA-LiP0 immunized group showed increased expression ratio of IFN-γ/IL-10 and IFN-γ/TGF-β in the lymph nodes before challenge. Two months after infection hamsters immunized with the empty plasmid presented a pro-inflammatory immune response in the early stages of infection with increased expression ratio of IFN-γ/IL-10 and IFN-γ/TGF-β, whereas hamsters immunized with pcDNA-HIS presented an increase only in the ratio IFN-γ/ TGF-β. On the other hand, hamsters immunized with LiP0 did not present any increase in the IFN-γ/TGF-β and IFN-γ/IL-10 ratio independently of the immunization strategy used. Conversely, five months after infection, hamsters immunized with HIS maintained a pro-inflammatory immune response (ratio IFN-γ/ IL-10) while pcDNA-LiP0 immunized hamsters continued showing a balanced cytokine profile of pro and anti-inflammatory cytokines. Moreover we observed a significant reduction in parasite load in the spleen, liver and lymph node in this group compared with controls.

**Conclusions/Significance:**

Our results suggest that vaccination with *L. infantum* LiP0 antigen administered in a DNA formulation could be considered a potential component in a vaccine formulation against visceral leishmaniasis.

## Author Summary

Visceral leishmaniasis caused by *Leishmania infantum* is the most severe form of leishmaniasis. The disease is fatal if not treated and there is no vaccine available for human use. In the search for potential antigens, the protective ability of conserved parasite protein families such as *L*. *infantum* histones (HIS) and acidic ribosomal (LiP0) antigens were successfully tested in the mouse model of cutaneous leishmaniasis. Here, we evaluate HIS and LiP0 antigens using two different immunization strategies in the hamster model of visceral leishmaniasis. Hamsters are highly susceptible to *L*. *infantum* infection and we demonstrate that immunization with LiP0, but not HIS, protects against the fatal outcome of visceral leishmaniasis. Immunization with LiP0 was able to induce an increased expression of IFN-γ in detriment of IL-10 and TGF-β in the draining lymph node before infection creating an inhospitable environment for parasite growth. Following challenge, a reduced parasite load in the lymph node, spleen and liver of LiP0 immunized hamsters was detected five months after challenge. These findings suggest that LiP0 used in a DNA formulation could be considered a potential component in a vaccine formulation against visceral leishmaniasis.

## Introduction

Leishmaniasis is a parasitic disease caused by protozoan from the genus *Leishmania* transmitted by the bite of infected sand flies. Leishmaniasis is one of the six major tropical diseases targeted by the World Health Organization [1]. The disease has a broad spectrum of clinical manifestations, from cutaneous, self-limited skin lesions to a visceral form of the disease. Visceral leishmaniasis (VL) caused by *Leishmania infantum* in the New World is the most severe form of disease characterized by hepatosplenomegaly, fever with a high mortality rate if not treated [2].

Although extensive research has been performed to identify an antigen able to elicit a long lasting protection against infection, there is still no successful vaccine available for human leishmaniasis. The majority of *Leishmania* vaccine candidates tested are able to induce humoral and/or cellular immune responses. However, the immune response derived is not able to induce protection and may contribute to pathology exacerbation [1].

Several secreted and surface *Leishmania* antigens have been tested, targeting virulence factors or molecules important for parasite invasion but most of these candidates resulted in a short-lived or partial protection [3]. On the other hand, intracellular house-keeping proteins are able to modulate the host immune response because they do not undergo selective pressure by the immune response [4]. During infection, these molecules are released after the destruction of intracellular amastigotes by activated macrophages but they can be also excreted by non-classical secretion pathways [5,6]. Several intracellular antigens such as heat shock proteins, ribosomal proteins and histones have been investigated as potential vaccine candidates against different species of *Leishmania* [3]. Histones (HIS) are important structural proteins in the organization and regulation of genes. There are four main classes of histones (H2A, H2B, H3, and H4) that are responsible for the composition of the *Leishmania* nucleosome [7]. Iborra *et al* demonstrated that immunization with DNA plasmid coding for nucleosomal histones plus CpG adjuvant was able to significantly reduce lesion size after challenge with *L*. *major* in BALB/c mice [8]. More recently, Carneiro *et al*. observed that mice immunized with either a plasmid DNA cocktail composed of four different *Leishmania* histones or with a combination of the DNA cocktail followed by the corresponding recombinant proteins, resulted in the absence of infected macrophages at the site of challenge with *L*. *braziliensis* in the presence of sand fly saliva. The protective response was associated with increased expression of IFN-γ and down regulation of IL-4 at the infection site [9].

Another immunodominant antigen is the *Leishmania infantum* acidic ribosomal protein (LiP0), a structural component of the large ribosome subunit that has been recognized by sera from both patients and dogs infected by *L*. *infantum* [10,11]. Also, it has been demonstrated that ribosomal protein (P0) was able to stimulate proliferation and IFN-γ secretion of a T-cell clone established from a human donor by stimulation with paraformaldehyde fixed promastigotes [12]. Using an immunization strategy that included LiP0 plasmid DNA vaccination and/or with LiP0 recombinant protein plus CpG, Iborra *et al*. showed the induction of partial protection against *L*. *major* infection in BALB/c mice [13]. However, C57BL/6 mice immunized with the same strategy was able to significantly reduce parasite load controlling lesion development [14]. The induction of IFN-γ was related with protection against *Leishmania* infection in these models. Although not demonstrated in *Leishmania*, it has been shown that P0 can be located in the surface of some protozoan parasites from the genus *Plasmodium* and *Toxoplasma* [15]. Its location on the cell surface of these organisms underlies the protective responses elicited by different vaccines based on P0 antigen. Thus, the generation of humoral responses to *Neospora caninum* P0 induces protection against neosporosis and toxoplasmosis [16]. Similarly, an experimental vaccine based on the carboxy-terminal domain of *Plasmodium falciparum* P0 was able to induce humoral responses that protects mice against malaria [17].

However, the immunization strategy used can be imperative to improve immunogenicity. The heterologous prime-boost strategy is used combining different formulations of the same antigen and has been effective in immunizations against cutaneous and visceral leishmaniasis. Dogs immunized with LACK using the heterologous strategy (DNA/protein) presented protection against VL caused by *L*. *infantum* [18]. Similarly, Iborra *et al*. (2005) used the same strategy with pcDNA3-LiP0 followed by recombinant protein (rLiP0) that protected C5BL/6 mice against challenge with *L*. *major* [14]. Moreover, administration of CpG ODN with antigens from different pathogens has been shown to induce a strong Th1 immune response [19].

Therefore, based on the protective potential of antigens belonging to conserved protein families used in the cutaneous leishmaniasis mouse model, we hypothesized that immunization with HIS and LiP0 antigens could also protect hamsters against the fatal outcome of *L*. *infantum* infection. In addition, we also compared HIS and LiP0 antigens using two different immunization strategies: plasmid DNA only (homologous) or plasmid DNA and recombinant protein plus CpG ODN (heterologous).

## Methods

### Animals

Male Syrian golden hamsters (*Mesocricetus auratus*) six to eight weeks old were obtained from Centro de Pesquisa Gonçalo Moniz/ Fundação Oswaldo Cruz (FIOCRUZ) animal facility. All animal work was conducted according to the Guidelines for Animal Experimentation of the Colégio Brasileiro de Experimentação Animal and of the Conselho Nacional de Controle de Experimentação Animal. The local Ethics Committee on Animal Care and Utilization (CEUA) approved all procedures involving animals (CEUA—Centro de Pesquisas Gonçalo Muniz—CPqGM/FIOCRUZ—L-IGM-011/09 and 005/2011.

### DNA plasmids coding for LiP0 and nucleossomal histones

DNA plasmid coding for *Leishmania infantum* acid ribosomal protein (pcDNALiP0) and the histones pcDNAHIS (pcDNA3LiH2-H3 and pcDNA3LiH2B-H4) were cloned and purified as previously described [13,14].

### Immunization strategies

Immunization experiments were carried out in groups of 15 hamsters. In the homologous immunization experiments, hamsters were inoculated three times intramuscularly (i.m.) with 100 μg of DNA of pcDNA3-LiP0, pcDNA3 HIS (50 μg of each plasmid) or pcDNA3 plus 1nM of CpG ODN 1826 (18–24 pb—5´TCC ATG ACG TTC CTG ACG TT-3´ mol wt 6364,1g/mol) (empty plasmid) in a total volume of 50 μL. In the heterologous strategy, hamsters received two inoculations of DNA followed by one intradermal (i.d.) inoculation of recombinant protein (5 μg of each rHIS or 10 μg rLiP0 protein) plus 1 nM of CpG ODN 1826 in the right ear [20]. In all groups, hamsters were inoculated at 2-week intervals. Two weeks after the last immunization 15 hamsters per group were euthanized to collect draining lymph nodes and sera to evaluate the cellular and humoral response induced by the immunization.

### Determination of antibody titers

Fifteen days after last immunization and two and 5 months after infection a sample of blood was collected from 6–8 hamsters per group by retrorbital plexus and sera was obtained after centrifugation for 1500 rpm for 5 minutes and stored at-4°C until use. Briefly, to measure specific antibody responses by ELISA, standard ELISA plates were coated overnight at room temperature with 100μL of SLA (10 μg/mL), rLiP0 (2μg/mL) or rHIS (1 μg/mL) in PBS. Serum samples were added at dilutions of 1:100. Following a washing step, a goat anti-hamster IgG alkaline phosphatase conjugate (1:1,000 and 1:2,000, Sigma, Missouri, USA) was added and incubated for one hour. The wells were then re-washed, substrate and chromogen (p-nitrophenyl phosphate; Sigma, USA) were added, and absorbance was recorded at 405 nm on a SpectraMax 190 spectrophotometer (Molecular Devices, USA) automatic micro plate reader.

### Intradermal challenge with *Leishmania infantum*

*Leishmania infantum* (MCAN/BR/00/BA262) promastigotes isolated from a naturally infected dog (Bahia State, Brazil) were cultured in Schneider’s medium (LGC, Brazil) supplemented with 10% of inactivated FBS (fetal bovine serum) (Gibco, USA), 2 mM L-glutamine, 100 IU/ml penicillin, 1% streptomycin (Gibco, USA).

Fifteen days after the last immunization, hamsters were inoculated by intradermal route, in the left ear with 105 stationary phase promastigotes plus 0.5 pair of sonicated salivary glands (SGH) from female *Lutzomyia longipalpis*, using a 29-gauge needle (BD Ultra-Fine) in 20uL of saline. Salivary glands were dissected from 5- to 7-day-old females and stored in endotoxin-free PBS at −70°C. Salivary glands were sonicated (Sonifer 450 homogenizer, Branson, Danbury, Connecticut), and afterwards centrifuged at 12,000 g for 5 minutes. Supernatant was collected and used immediately.

### Limiting Dilution Assay (LDA)

Parasite load was determined 2 and 5 months post-infection using the quantitative Limiting Dilution Assay (LDA) as described by Titus et al [21]. Briefly, infected liver, spleen and retromaxillar draining lymph nodes were aseptically removed from individual hamsters. Tissues were homogenized and diluted in Schneider’s Insect Medium (Sigma, St. Louis, MO) supplemented with 10% heat inactivated fetal bovine serum (Gibco, USA), 100 U of penicillin/ml and 100 mg/ml of streptomycin. Homogenate samples were serially diluted into 96-wells plates containing biphasic blood agar (Novy-Nicolle-McNeal) medium and incubated for one week at 23°C when the presence of viable parasites was determined. Parasite burden in the tissue was calculated applying ELIDA software [21].

### RNA isolation and quantitative Real-Time PCR

Using Trizol reagent (Invitrogen, USA), total RNA was extracted from the draining retromaxillar lymph nodes two weeks after the last immunization, and from the spleen obtained two and five weeks after infection. First-strand cDNA synthesis was performed with 1–2 μg of RNA in a total volume of 25 μL using Super Script II (Gibco, Carlsbad, CA, USA). DNA was amplified in the thermocycler (Mastercycler gradient—Eppendorf, USA) with an initial pre- incubation at 72°C for 5 minutes, followed by amplification of the target DNA at 42°C for 50 minutes. A standard curve was generated for each set of primers and efficiency of each reaction was determined. The expression levels of genes were normalized to GAPDH levels. Results are expressed in fold change over control. Oligonucleotide primers used were: GAPDH (reverse 5’- CTGACATGCCGCCCTGGAG-3’ and forward 3’-TCAGTG- TAGCCCAGGATGCC-5’); IFN-γ (reverse 5’-GAAGCTCAC- CAAGATTCCGGTAA-3’ and forward 3’-TTTTCGTGACA- GGTGAGGCAT-5’); IL-10 (reverse 5’-AGACGCCTTTCTC- TTGGAGCTTAT-3’ and forward 39-GGCAACTGCAGCGC- TGTC-5’); and TGF-β (reverse 5’-GCTACCACGCCAACTTC- TGTC-3’ and forward 3’-TGTTGGTAGAGGGCAAGG-5’).

### Histology

For histology, five months after infection spleen and liver fragments of four hamsters from each group were fixed in 10% phosphate-buffered formalin and embedded in paraffin. Four-micrometer sections were stained with hematoxylin–eosin and studied by optical microscopy.

### Statistical analysis

Experiments were repeated three times with five hamsters per group per time point. Comparisons among immunized and non-immunized control groups were done by one-way ANOVA (Kruskal-Wallis) analysis with Dunn’s post-test. Results were considered statistically significant when p≤0.05. All statistical analysis was done using Graph Pad 5.0 software program.

## Results

### Heterologous immunization strategy using HIS or LiP0 antigens induces production of specific antibodies

**Fig 1:**
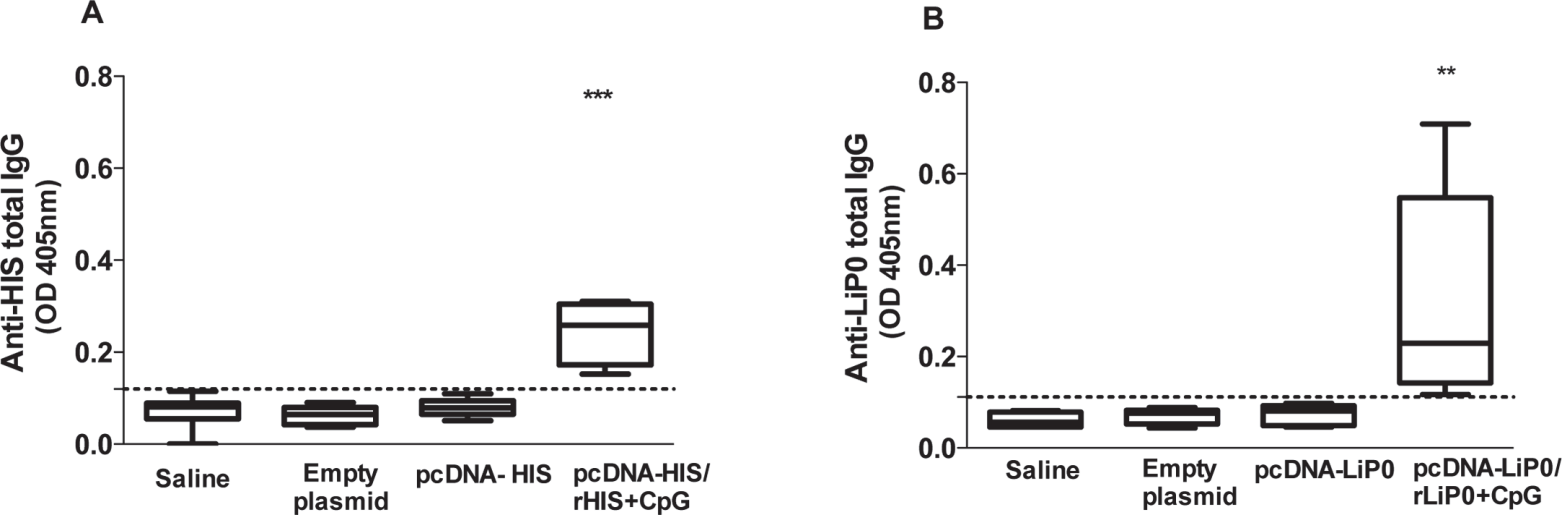
Humoral immune response after immunization with nucleosomal histones (HIS) or acidic ribosomal protein (LiP0) antigens. Hamsters (n = 6–10) were bled 2 weeks after immunization and sera were tested by ELISA to determine specific total IgG levels. (A) Anti-HIS total IgG in sera from hamsters immunized with recombinant DNA coding for HIS (pcDNA HIS) or with recombinant DNA followed by recombinant nucleosomal histones+CpG (pcDNA HIS-rHIS+CpG). (B) Anti-LiP0 total IgG in sera from hamsters immunized with recombinant DNA coding for LiP0 (pcDNA LiP0) or with recombinant DNA followed by recombinant protein LiP0+CpG (pcDNA LiP0- rLiP0+CpG). Control groups were immunized with saline or empty plasmid. *p<0.05; **p<0.01; ***p<0.001, compared with controls.

To evaluate production of anti-HIS or anti-LiP0 IgG antibodies we collected sera from immunized hamsters fifteen days after the last immunization. We observed that only hamsters that were immunized with heterologous strategy showed significantly higher titers of antibodies. There was a significant increase of IgG in the group that received DNA plasmid coding for HIS followed by booster of nucleosomal histone recombinant protein plus CpG adjuvant (pcDNA HIS-rHIS+CpG) compared to non-immunized hamsters (Fig. 1A). Similar results were observed regarding antibody production in hamsters immunized with LiP0, using the same heterologous strategy. There was a significantly higher production of anti-LiP0 IgG in vaccinated hamsters (pcDNA-LiP0/ rLiP0+CpG) compared to the animals that received empty plasmid and saline, the control groups (Fig. 1B).

### Immunization with DNA plasmid coding HIS and LiP0 proteins increase expression of IFN-γ in the lymph node

**Fig 2:**
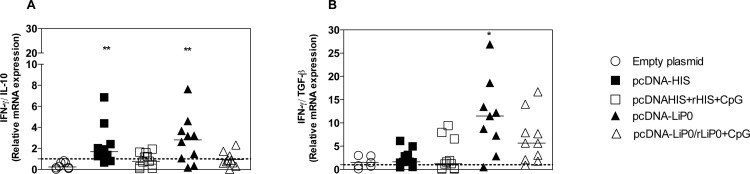
Cytokine expression following immunization with nucleosomal histones (HIS) or acidic ribosomal protein (LiP0) antigens. Hamsters (n = 6–10) were immunized with HIS or LiP0 antigens using the homologous (DNA only) or heterologous (DNA-recombinant protein plus CpG) and retromaxillar lymph nodes collected for evaluation of cytokines expression by Real-Time PCR, two weeks after the last immunization. (A) IFN-γ/IL-10 mRNA expression ratio and (B) IFN-γ/TGF-β mRNA expression ratio. *p<0.05; **p<0.01; ***p<0.001, compared with controls.

In order to verify the cellular immune response induced by the immunization with HIS and LiP0, expression of IFN-γ, IL-10, and TGF-β was evaluated in retromaxillar draining lymph nodes 15 days after immunization. We observe a significant increase in the ratio of IFN-γ/IL-10 expression in the lymph nodes of animals immunized with HIS and LiP0 using the homologous immunization strategy with DNA plasmids (Fig. 2A). Conversely, there was no difference in the IFN-γ/TGF-β ratio in the HIS immunized group using both immunization strategies (Fig. 2B). Interestingly, we observed that the IFN-γ/TGF-β ratio was significantly higher in hamsters immunized with DNA plasmid (pcDNA-LiP0) using homologous strategy compared with the control group (Fig. 2B).

### Immunization with LiP0 protects hamsters against the fatal outcome of visceral leishmaniasis

In order to verify if the immune responses resulting from immunization with acidic ribosomal protein and nucleosomal histone antigens using homologous and heterologous immunization strategies, are able to protect against *L*. *infantum* infection, immunized hamsters were challenged with 10^5^ *L*. *infantum* plus SGH, trying to mimic natural transmission [22].

**Fig 3:**
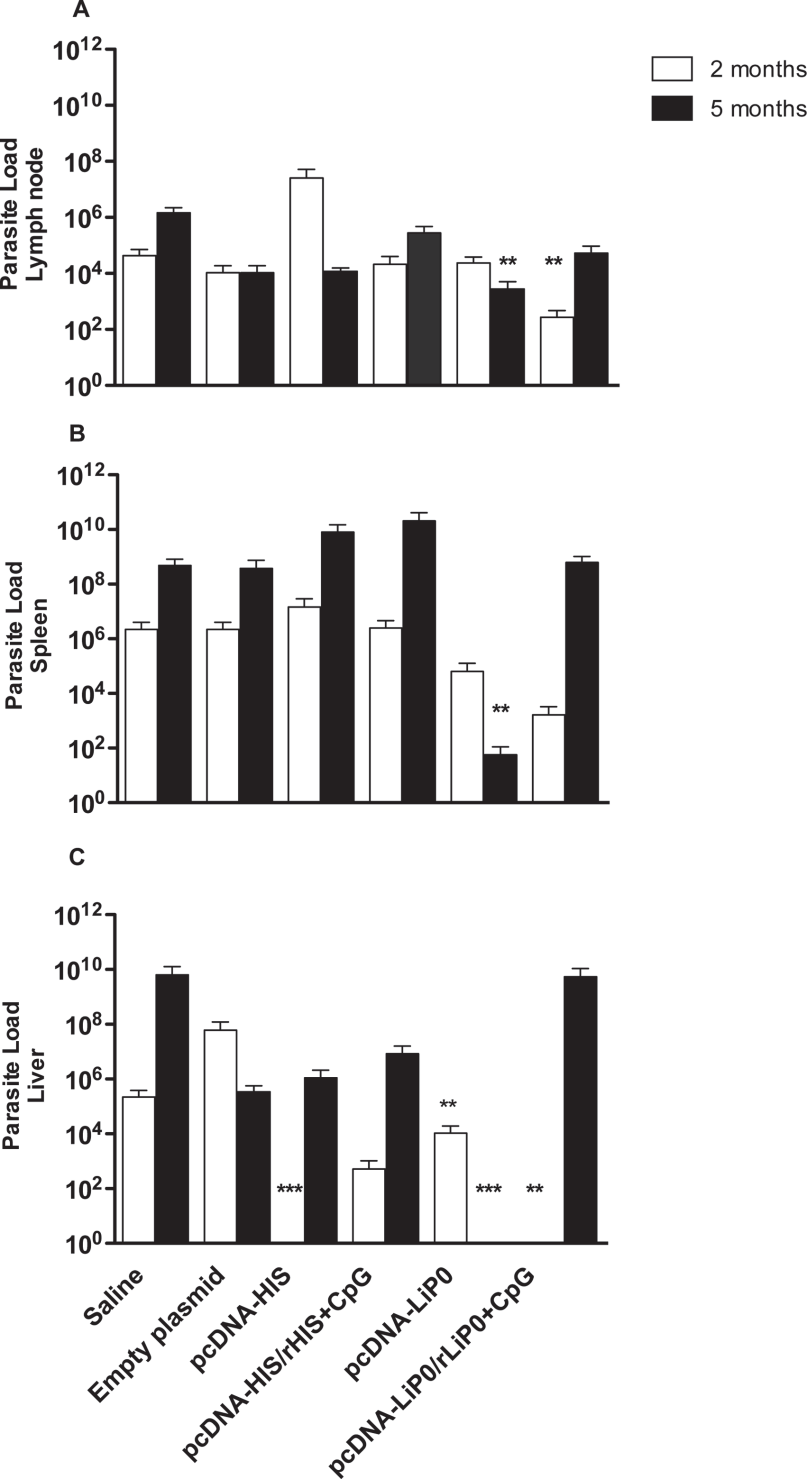
Parasite load in hamsters immunized with HIS or LiP0 antigens following *L. infantum* infection. Hamsters (n = 15) were immunized with HIS or LiP0 antigens using the homologous (DNA only) or heterologous (DNA-recombinant protein plus CpG). Two weeks after the last immunization, hamsters were challenged in the left ear with 10^5^ *L*. *infantum* in the presence of *Lu*. *longipalpis* SGH. Draining retromaxillar lymph node (A), Spleen (B) and Liver (C) parasite loads per total organ were determined two and five months after challenge by limiting dilution assay (LDA). *p<0.05; **p<0.01; ***p<0.001, compared with controls.

Two months after challenge, hamsters immunized with HIS using the homologous strategy showed a significant parasite load reduction in the liver (Fig. 3C) but not in the retromaxillar lymph nodes or spleen compared to control groups (Fig. 3A and B).

On the other hand, we observed a significant reduction in the lymph node, spleen and liver parasite loads in LiP0 DNA plasmid (pcDNA3-LiP0) immunized group, compared with controls five months after challenge (Fig. 3A, B and C). Interestingly, parasite burden in the spleen (Fig. 3B) (p = 0.0016) and liver (Fig. 3C) (p = 0.0001) of hamsters immunized with the LiP0-based DNA vaccine were lower at 5 months than 2 months after infection, indicating that the vaccine was able to induce a long term leishmanicidal response.

### Antibody levels in HIS and LiP0 immunized hamsters after *L.infantum* infection

To access the humoral immune response after infection, we measured production of anti-LiP0, HIS and SLA IgG antibodies. For this purpose, we collected sera from infected hamsters at two and five months following challenge with *L*. *infantum* plus SGS.

**Fig 4:**
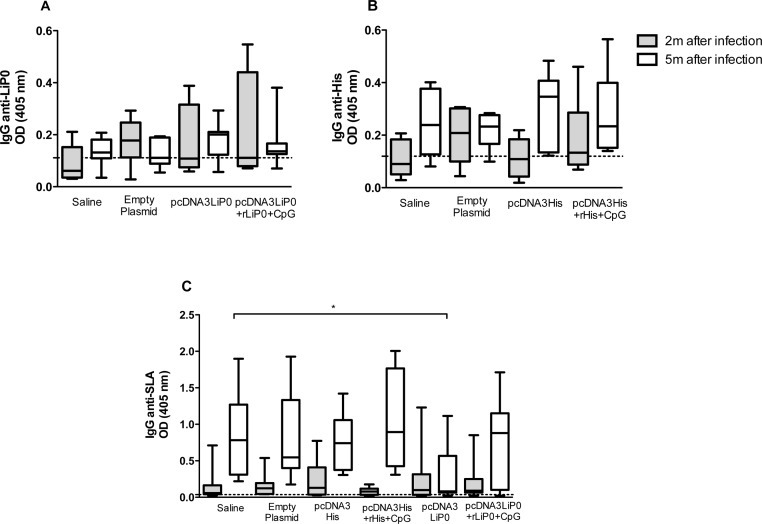
Humoral immune response in HIS or LiP0 immunized hamsters following *L. infantum* infection. Hamsters (n = 15) were immunized with HIS or LiP0 antigens using the homologous (DNA only) or heterologous (DNA-recombinant protein plus CpG). Two weeks after the last immunization, hamsters were challenged in the left ear with 10^5^ *L*. *infantum* in the presence of *Lu*. *longipalpis* SGH. Two and five months after challenge sera were tested by ELISA to determine specific total IgG levels. (A) Anti-HIS total IgG in sera from hamsters immunized with pcDNA HIS or with pcDNA HIS-rHIS+CpG. (B) Anti-LiP0 total IgG in sera from hamsters immunized with DNA LiP0 or DNA LiP0-rLiP0+CpG. (C) Anti-SLA total IgG in sera from hamsters immunized with HIS or LiP0. *p<0.05; **p<0.01; ***p<0.001, compared with controls.

We observed an increase in anti-HIS IgG in all groups immunized with HIS especially at five months after infection (Fig. 4A). Conversely, LiP0 immunized groups did not present any significant levels of anti-LiP0 IgG levels two and five months following infection when compared with controls (Fig. 4B).

Interestingly, animals immunized with LiP0 homologous strategy showed a significantly lower anti-SLA IgG levels at five months after infection compared to HIS immunized and controls groups (Fig. 4C).

### Cytokines detection in HIS and LiP0 immunized hamsters after *L*. *infantum* infection

To evaluate the cellular immune response of hamsters challenged with *L*. *infantum*, we investigated the expression of cytokines in the spleen two and five months following infection.

**Fig 5:**
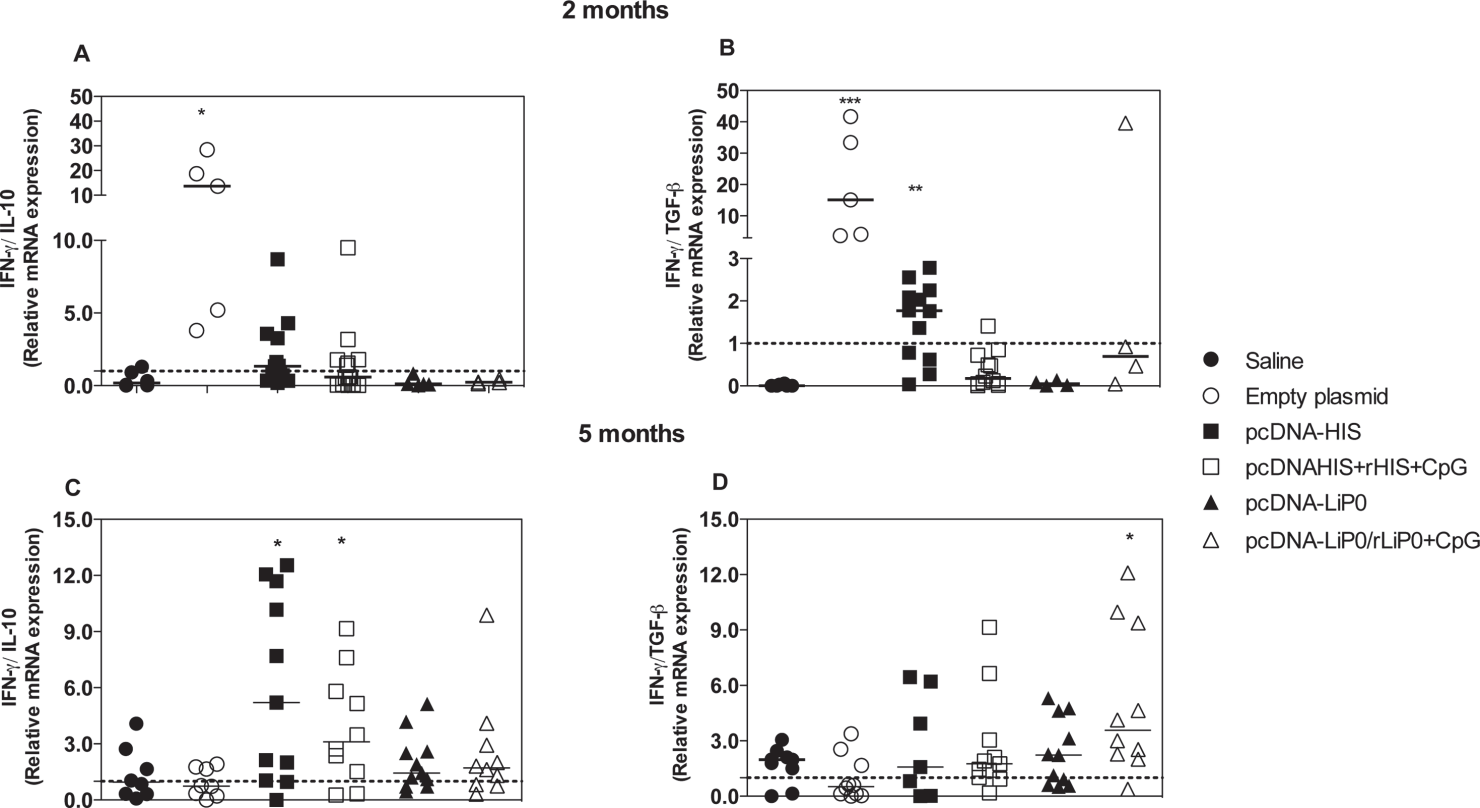
Cytokine expression in the spleen of HIS or LiP0 immunized hamsters following *L. infantum* infection. Hamsters (n = 4–11) were immunized with HIS or LiP0 antigens using the homologous (DNA only) or heterologous (DNA-recombinant protein plus CpG). Two weeks after the last immunization, hamsters were challenged in the left ear with 10^5^ *L*. *infantum* in the presence of *Lu*. *longipalpis* SGH. Spleen samples were collected for evaluation of mRNA expression of IFN-γ, IL-10 and TGF-β by Real-Time PCR. IFN-γ/IL-10 mRNA expression ratio at two (A) and five (C) months after challenge; and IFN-γ /TGF-β mRNA expression ratio at two (B) and five (D) months after challenge. *p<0.05; **p<0.01; ***p<0.001, compared with controls.

Two months after challenge hamsters immunized with DNA-HIS (homologous strategy) presented an increased expression ratio of IFN-γ/IL-10 when compared with the non-immunized control group (p = 0.0253) (Fig. 5A). Expression ratio of IFN-γ/TGF-β was increased in the pcDNA-HIS (p = 0.0003), and pcDNA-HIS/rHIS+CpG (p = 0.018) immunized groups compared with saline control suggesting that DNA-HIS immunized hamsters presented a pro-inflammatory immune response in the early stages of infection (Fig. 5B). On the other hand, hamsters immunized with LiP0 using independent of strategy employed did not present any increase in the expression ratio of IFN-γ/IL-10 or IFN-γ/TGF-β (Fig. 5A and B).

Five months after infection, hamsters immunized with HIS using both immunization strategies showed an increased expression ratio of IFN-γ/IL-10 (p<0.05) suggesting a pro-inflammatory immune response (Fig. 5C). Concerning LiP0 immunization, there was an increase in the expression ratio of IFN-γ/TGF-β in the spleen of pcDNA-LiP0/rLiP0+CpG immunized hamsters (heterologous strategy) compared with the control group (p<0.0101) (Fig. 5D) suggesting that this strategy induce a delayed cellular immune response.

### The spleen and liver of LiP0 immunized hamsters present fewer granulomas and macrophage aggregates

**Fig 6:**
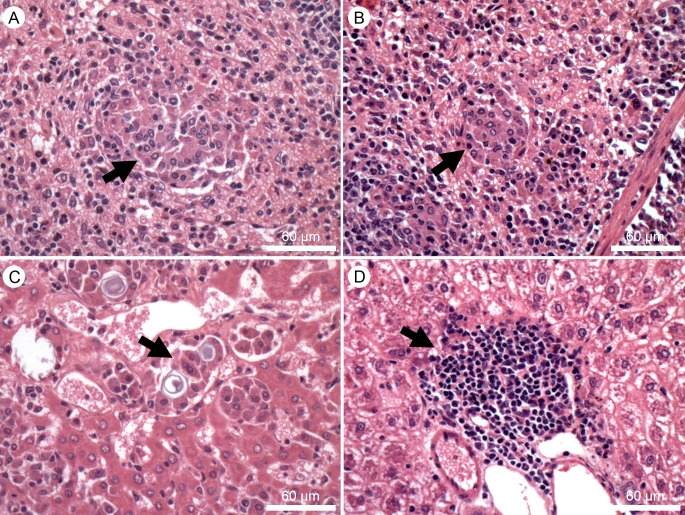
Histological aspects of hamsters immunized with LiP0 antigen following *L. infantum* infection. Hamsters (n = 4) were immunized with HIS or LiP0 antigens using the homologous (DNA only) or heterologous (DNA-recombinant protein plus CpG) immunization strategies. Two weeks after the last immunization, hamsters were challenged in the left ear with 10^5^ *L*. *infantum* in the presence of *Lu*. *longipalpis* SGS. Five months after challenge, spleen (A and B) and liver (C and D) were obtained, from control (A and C) and LiP0 (B and D) immunized hamsters, fixed and stained with H&E for histological evaluation.

Five months after infection spleen and liver samples were collected from four hamsters per group for histopathological analysis. In the spleen, we observed disorganized follicles with or without germinal center, macrophage aggregates with *L*. *infantum* amastigotes inclusions, epithelioid cells present in the red and white pulp, as well as granulomas in the groups immunized with HIS or LiP0. However, hamsters immunized with HIS displayed a higher loss of splenic structure… Animals immunized with LiP0 presented smaller macrophage aggregates. Fig. 6 shows the granulomas present in the control (A) and LiP0 (B) immunized hamsters after infection (arrows).

At the same time point, liver from control and HIS immunized groups showed accentuated intrasinusoidal leukocytosis with portal mononuclear infiltrate. Areas of necrosis and granulomas containing giant cells filled with inclusions of amorphous material, few Schaumann bodies were observed (arrows in Fig. 6C). Although HIS and LiP0 immunized groups presented comparable intense inflammatory infiltrate the majority of hamsters immunized with LiP0 antigens presented fewer granulomas (arrows in Fig 6D). These data were summarized in Table 1, showing the number and percentage of animals displaying the histopathological alterations.

**Table 1:** Spleen and liver histopatholgical alteration after five months of infection in hamsters immunized with different strategies.

Spleen	Folicles without germinal center	Folicles with germinal center	Follicles with atrophy	Granuloma	Macrophage aggregates
Saline	1/4 (25%)	0/4 (0%)	2/4 (50%)	0/4 (0%)	0/4 (0%)
Empty Plasmid	1/4 (25%)	1/4 (25%)	0/4 (0%)	1/4 (25%)	0/4 (0%)
pcDNA-HIS	0/4 (0%)	2/4 (50%)	0/4 (0%)	2/4 (50%)	0/4 (0%)
pcDNA-HIS/rHIS+CpG	0/4 (0%)	4/4 (100%)	0/4 (0%)	1/4 (25%)	1/4 (25%)
pcDNA-LiP0	0/4 (0%)	4/4 (100%)	0/4 (0%)	2/4 (50%)	0/4 (0%)
pcDNA-LiP0/rLiP0+CpG	0/4 (0%)	3/4 (75%)	0/4 (0%)	3/4 (75%)	0/4 (0%)
Liver	Leukocytosis	Portal mononuclear infiltrate	Inclusions of amorphous material	Granuloma	Macrophage aggregates
Saline	2/4 (50%)	2/4 (50%)	0/4 (0%)	1/4 (25%)	1/4 (25%)
Empty Plasmid	3/4 (75%)	3/4 (75%)	0/4 (0%)	3/4 (75%)	0/4 (0%)
pcDNA-HIS	2/4 (50%)	0/4 (0%)	2/4 (50%)	2/4 (50%)	1/4 (25%)
pcDNA-HIS/rHIS+CpG	3/4 (75%)	3/4 (75%)	0/4 (0%)	2/4 (50%)	0/4 (0%)
pcDNA-LiP0	2/4 (50%)	3/4 (75%)	0/4 (0%)	1/4 (25%)	0/4 (0%)
pcDNA-LiP0/rLiP0+CpG	1/4 (25%)	3/4 (75%)	0/4 (0%)	1/4 (25%)	0/4 (0%)

## Discussion

*Leishmania* nucleosomal histones (HIS) and acidic ribosomal protein P0 (LiP0) elicit defined immune responses and have been the focus of investigation as potential vaccines against cutaneous leishmaniasis [3]. Herein, we further explore their antigenic potential against the visceral form of leishmaniasis. Using the susceptible hamster model of VL we show that both molecules are immunogenic but only immunization with LiP0 is able to control disease progression.

Following immunization a notable difference was already observed on the profile of cytokine expression in the draining lymph nodes. The expression ratio of IFN-γ/IL-10 of hamsters immunized with pcDNA-LiP0 and pcDNA-HIS was significantly higher compared with control groups. Interestingly, significant differences in the expression ratio of IFN-γ/TGF-β was observed only in hamsters immunized with LiP0 using the homologous strategy. Similar results were observed previously where production of IFN-γ but not IL-4 was detected in the spleen of mice immunized with nucleosomal HIS antigens [8]. Another study also demonstrated IFN-γ production upon stimulation of splenocytes and lymph node cells *in vitro* with rLiP0 after immunizations with pcDNA-LiP0 [13].

In the group immunized with empty vector we detected higher ratios of IFN-γ /IL-10 and IFN-γ/TGF-β two months after infection, suggesting that the infection could be responsible for this increase. Additionally, we did not detect the same levels for the control group (saline). The empty vector contains CpG motifs that inespecifically increase production of inflammatory cytokines, such as IFN-γ. It is important to emphasize that evaluation of cytokines in the spleen was done *ex vivo* that does not comprise specific *in vitro* restimulation. Therefore, we can speculate that the higher number of parasites present in the spleen could contribute to the inflammatory pattern observed. However, IFN-γ expression was not sustained, five months after infection cytokines ratios returned to basal levels that correlated with a high parasite load in the liver and spleen. The same interpretation could be applied to the group immunized with HIS, concerning the restimulation *in vitro*, where at two months, immunization with HIS induce a lower IFN-γ/IL-10 ratio. However, five months after infection, the number of parasites was high in the spleen and possibly, in an attempt to control parasite growth, there was an increase in IFN-γ expression as observed in other reports in the literature [22]. Interestingly, the ratio of IFN-γ/TGF-β presented opposite results, where we observed an increase in this ratio at two months post-infection and a decrease five months after infection. We know that IL-10 and TGF-β play similar roles in the inhibitory responses and we can speculate these cytokines might alternate in the balance of pro and anti-inflammatory responses after infection [23,24].

Moreover, the expression ratio of IFN-γ/IL-10 and IFN-γ/TGF-β in the animals immunized with LiP0 antigens did not present any significant increase, independently of the immunization strategy used. Interestingly, these animals displayed a significant reduction in the parasite load in lymph nodes, spleen and liver five months after infection, suggesting that this modulation in the immune response is important for infection control. Hamsters immunized with LiP0 also presented fewer infected granulomas and macrophage aggregates in the spleen and liver and lower anti-SLA IgG levels after infection compared to the HIS immunized group indicating control of parasite replication.

A significant production of IgG antibodies was also detected following immunization with both antigens (LiP0 or HIS). Similarly, Iborra *et al*. also detected specific levels of IgG and IgG2a in mice immunized with LiP0 and HIS antigens. Antibodies against a LiP0 ribosomal protein epitope were also detected in dogs naturally infected with *L*. *infantum* [8,11,14]. Specific total IgG production was only detected when the heterologous immunization strategy was employed. Indeed, vaccination strategies using exclusively DNA are known to elicit low IgG production as previously demonstrated [25]. Interestingly, five months after infection, anti-HIS and anti-LiP0 IgG levels increased in all groups with no significant differences between immunization strategies.

The significant parasite load reduction observed in the pcDNA-LiP0 immunized group after five months could be a result of the increased expression ratio of IFN-γ/IL-10 and IFN-γ/TGF-β in the lymph nodes before challenge. The induction of a predominant Th1 immune response following immunization could have an impact on parasite establishment and disease development. The importance of a Th1 environment established before challenge was clearly demonstrated in hamsters immunized with LJM19, a vector salivary molecule, Protection was associated with a considerably higher expression of IFN-γ/TGF-β ratio in the spleen compared with controls [22].

Using the mouse model, Carrion *et al*. demonstrated that immunization with a pcDNA-HIS vaccine and subsequently challenged with *L*. *infantum* did not result in parasite load reduction [26]. Another study demonstrated different results with parasite load reduction in the spleen and liver of mice immunized with HIS pulsed dendritic cells associated with CpG after *L*. *infantum* infection. Protection was associated with a potent polarized Th1 type immune response elicited by the immunostimulatory ability of HIS pulsed dendritic cells [27]. More recently, immunization with recombinant histones proteins from *L*. *donovani* was able to confer protection against *L*. *donovani* infection [28].

In the cutaneous leishmaniasis model, the protective ability of HIS was demonstrated using both homologous and heterologous immunization strategies. BALB/c mice immunized with HIS, using both experimental approaches and challenged with *L*. *braziliensis*, were able to control lesion development, decreased parasite burden in lymph nodes and absence of infected macrophages at site of infection [9]. In another study, DNA-HIS vaccines were able to reduce the number of *L*. *major* parasites in the draining lymph node and spleen compared to controls [8]. Contrasting results between different animal models and clinical forms were also observed after immunization with KMP11, a different parasite antigen. Immunization of hamsters with KMP11 was able to confer protection against *L*. *donovani* and *L*. *infantum* infection. The same protective response was not observed when encapsulated KMP11 was used to immunize BALB/c mice against *L*. *braziliensis* infection [29–31]. These contradictory findings concerning visceral and cutaneous leishmaniasis models could be explained by the remarkable differences in disease development and the host’s immune response depending on the animal model used. Visceral leishmaniasis is a systemic chronic disease that affects many organs while cutaneous leishmaniasis is characterized by a restricted skin lesion. Although the mouse model has been extensively used to study cutaneous disease, infection with *L*. *infantum* is self-limiting. The hamster model of VL is a well described model of susceptibility, with progressive disease that more closely mimics the severity of infection in humans and dogs [32,33]. Assessment of long term memory is important when evaluating a potential vaccine candidate. Unfortunately, there are no immunological tools available to investigate and characterize memory cells in hamsters.

Vaccines based on combination of different antigen candidates have been shown to improve protection. In fact, a recombinant Q protein formed by genetic fusion of five parasite intracellular antigens has been successfully tested in dogs [34–36]. Although this is imperative when considering a new vaccine, the focus of this study was to initially test HIS and LiP0 antigens independently. Besides that, combination of vector salivary antigens, such as LJM19, and parasite antigens could improve protection compared to immunization with isolated antigens. In order to select the antigens that could be used in combination its necessary to initially evaluate their immunogenicity and protection potential independently. Interestingly, more recent data published by our group have showed that combination of LJM19 plasmid with KMP11, a parasite antigen, was not able to improve protection resulting from immunization with LJM19 or KMP11 alone in the hamster model[37].

There are two licensed vaccines for canine VL available in Brazil. The fucose mannose ligand of *Leishmania donovani* (Leishmune) [38,39] and the *Leishmania* amastigote recombinant A2-antigen (Leish-Tec) [40]. In a recent study, no significant differences in the rate of seroconversion, clinical signs, parasitism and parasite transmission to the vector were observed in dogs vaccinated with any of the two vaccines [41]. Although both vaccines showed promising results, large-scale field studies are necessary to validate their inclusion in a mass control strategy for canine VL.

Taken together, the data shown herein indicates that although *L*. *infantum* LiP0 and HIS antigens were immunogenic in hamsters, only LiP0 antigen was able to confer a significant degree of protection against *L*. *infantum* using the susceptible hamster model of VL. Importantly, we also noted that the immunization strategy used is critical when a potential vaccine candidate is being tested. Indeed, immunization with pcDNA-LiP0 followed by rLiP0 boost (heterologous strategy) resulted only in short-term protection after two months. Our results suggest that *L*. *infantum* LiP0 antigen administered in a DNA formulation (homologous strategy) is a potential vaccine candidate and further investigation of the protective mechanism could improve *L*. *infantum* LiP0 protective effect against visceral leishmaniasis.
